# Depth Prediction Improvement for Near-Field iToF Lidar in Low-Speed Motion State

**DOI:** 10.3390/s24248020

**Published:** 2024-12-16

**Authors:** Mena Nagiub, Thorsten Beuth, Ganesh Sistu, Heinrich Gotzig, Ciarán Eising

**Affiliations:** 1 Department of Front Camera, Valeo Schalter und Sensoren GmbH, 74321 Bietigheim-Bissingen, Germany; 2 Department of Electronic & Computer Engineer, University of Limerick, V94 T9PX Limerick, Ireland; ganesh.sistu@valeo.com (G.S.); ciaran.eising@ul.ie (C.E.); 3 Data-Driven Computer Engineering (D2iCE) Research Centre, University of Limerick, V94 T9PX Limerick, Ireland; 4 Department of Detection Systems, Valeo Detection Systems GmbH, 74321 Bietigheim-Bissingen, Germany; thorsten.beuth@valeo.com; 5 Department of Computer Vision, Valeo Vision Systems, NW1 186G Galway, Ireland; 6 Deptartment of Driving Assistance, Valeo Schalter und Sensoren GmbH, 74321 Bietigheim-Bissingen, Germany; heinrich.gotzig@valeo.com

**Keywords:** near field, Lidar, iTOF, depth correction, estimation, ambiguity, blurriness, motion blur, blind deconvolution, continuous learning

## Abstract

Current deep learning-based phase unwrapping techniques for iToF Lidar sensors focus mainly on static indoor scenarios, ignoring motion blur in dynamic outdoor scenarios. Our paper proposes a two-stage semi-supervised method to unwrap ambiguous depth maps affected by motion blur in dynamic outdoor scenes. The method trains on static datasets to learn unwrapped depth map prediction and then adapts to dynamic datasets using continuous learning methods. Additionally, blind deconvolution is introduced to mitigate the blur. The combined use of these methods produces high-quality depth maps with reduced blur noise.

## 1. Introduction

Automated driving systems face challenges in low-speed scenarios [1] due to the combination of static and slow-moving objects and crowded scenes, which complicates perception [2]. Sparse sensors like ultrasonic and short-range radars handle tasks like parking, while fisheye cameras provide broader views with dense depth data, requiring additional depth estimation [3,4]. Sensor fusion merges sparse and dense data, improving perception in such scenarios [3,5].

Emerging solutions like Indirect Time-Of-Flight (iTOF) lidar, such as Valeo’s Near-Field Lidars (NFLs) [6], offer dense depth information essential for low-speed driving. The Amplitude-Modulated Continuous Wave (AMCW)-based lidar [7] paired with CMOS 2D imagers [8,9] generates dense short-range point clouds, addressing key requirements.

However, iTOF technology faces depth wrapping issues, where depth measurements are constrained to specific depth cycles, creating ambiguous depth maps. This problem worsens in dynamic environments due to motion blur, which introduces noise and increases depth ambiguity. Addressing depth ambiguity and motion blur together is critical for advancing low-speed driving perception.

### 1.1. Contributions

State-of-the-art deep learning methods address depth unwrapping for static indoor scenes, where motion blur is not a concern. In contrast, other methods tackle motion blur in iTOF sensors without addressing depth unwrapping. However, motion blur significantly affects depth measurements; moving objects leave trailing traces at various depth locations, increasing ambiguity. This highlights a strong link between solving motion blur and efficient depth unwrapping. Notably, no existing approach addresses both depth wrapping and motion blur in a single task.

Our paper presents several contributions:

- We propose a new method to unwrap iTOF depth maps during motion and reduce motion blur artifacts, serving as a benchmark for future research. This method extends the NFL sensor depth range to the third and fourth depth cycles, enabling coverage up to 25 m, surpassing current methods limited to the second depth cycle.
- We introduce our new Atrous Spatial Inception Pyramid Module (ASIPM) to estimate the blur transformation of vertices for the overall context based on the study conducted by Huo et al. [10], a Visual Attention Network to estimate the blur transformation of vertices per specific objects, and an Inverse Blur Tensor Module to compensate for the effect of motion blur efficiently.
- Unlike existing approaches that require ambient light frames, our method exclusively uses laser measurements, making it ideal for visually challenging environments like smoke, dust, or darkness.

### 1.2. Paper Structure

Our paper is structured as follows: We begin with an introduction to the problem’s environment in Section 1. Then, in Section 2, we explain the problem and how motion complicates depth wrapping by introducing motion blur artifacts to the depth map. Section 3 explores the research already conducted to solve these problems. Following that, in Section 4, we explain our method to resolve the problems using a computer vision model. Section 5 presents our method’s results, comparing them to those of other state-of-the-art methods. Finally, in Section 6, we conclude and comment on the results.

## 2. Problem Description

The iTOF AMCW Lidar measures object distances by calculating the phase shift angle between transmitted and received laser signals [9]. This is achieved using multiple Differential Correlation Samples (DCSs) captured at varying phases, e.g., $0^{\circ }$, $90^{\circ }$, $180^{\circ }$, and $270^{\circ }$. The resulting phase shift angles matrix corresponds to the depth of each point in the point cloud. The depth *d* is calculated using (1) based on the phase shift angle $\varphi_{s}$ and the amplitude modulation frequency $f_{mod}$. When $\varphi_{s}$ is within 0 to 2π, the depth measurement is within the unambiguous range $d_{TOF}$. However, angles exceeding 2π are wrapped due to the sinusoidal nature of the phase shift, leading to a phase-wrapping effect that creates ambiguous depth maps.

(1) $$d_{TOF}=\frac{C}{2}\cdot \frac{\varphi_{s}}{2\pi f_{mod}}$$

Multi-modulation frequency methods [11] have been developed to unwrap ambiguous depth maps accurately. This technique captures groups of DCS frames at different modulation frequencies, typically using two frequencies. The resulting ambiguous depth maps are then compared to produce a final unwrapped depth map.

However, this method has drawbacks: it increases the active time of laser components, leading to potential overheating and reduced lifespan, and it raises the power consumption of the sensor device.

In iTOF sensors, motion causes the time-shifting of DCS frames, leading to motion blur in depth maps [8,9,12,13]. Unlike traditional camera motion blur, which results from light integration during long exposures [10,14], iTOF motion blur arises from the sensor’s operational principle [15].

**Figure 1:**
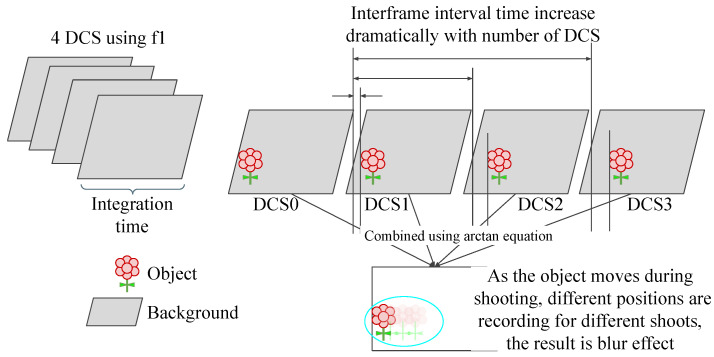
The motion blur effect occurs due to the correlation of four subsequent DCS frames. These four DCS frames are captured one after another, with an inter-frame interval time. When the sensor or environment moves, the object’s position in each DCS frame changes accordingly. Consequently, when the four DCS frames are correlated, a blurred image of the object is formed. The motion blur of the iTOF is unique because it appears in the form of blurred objects or trailing traces.

**Figure 2:**
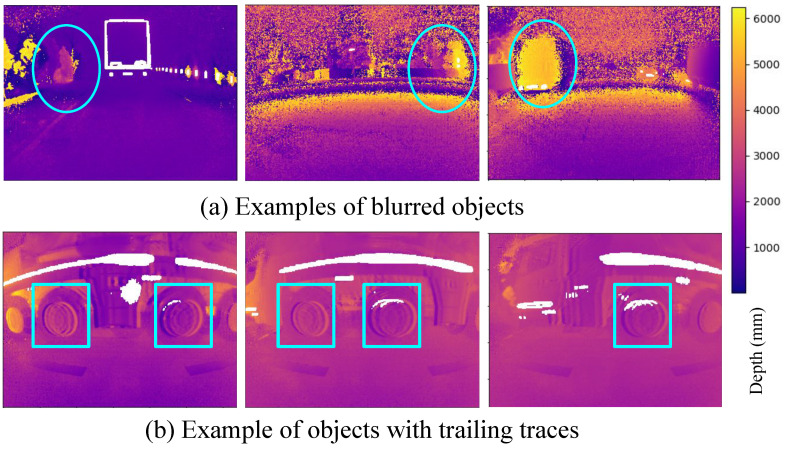
Samples from the dataset display the blur effect in the ambiguous depth maps. The top three frames depict the ambiguous depth maps of three different frames. The objects inside the ellipses are affected by motion blur noise. The bottom three frames illustrate ambiguous depth maps where objects inside the boxes are blurred and exhibit trailing traces. For instance, each vehicle wheel displays several trailing traces.

The sensor reconstructs depth maps by correlating multiple time-shifted DCS frames. In dynamic scenes, objects may change position between frames, causing the reconstructed point cloud to include trailing traces and motion blur. Figure 1 comprehensively explains the related motion blur case in iTOF sensors. Figure 2 illustrates this phenomenon and shows examples from our recorded dataset of these issues, highlighting a known limitation of iTOF sensors. Addressing this problem is critical for accurate depth correction.

The sensor’s CMOS imager captures grayscale images using ambient light alongside DCS frames. However, because the frames are captured sequentially in different time slots, time shifts may occur between the DCS frames and the grayscale image.

**Figure 3:**
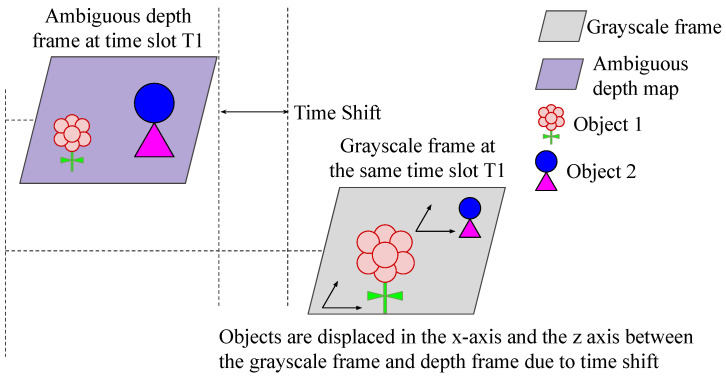
Due to a time shift between capturing the DCS samples forming the ambiguous depth map and the grayscale image, along with the sensor’s motion and changes in the environment, the size and position of each object change between the grayscale frame and the corresponding ambiguous depth map. This alteration causes a non-uniform spatial transformation of objects.

When the sensor or environment is in motion, the perspective viewport of the imager causes non-uniform spatial transformations of objects. This leads to spatial and dimensional changes when comparing the ambiguous depth frame with the grayscale frame. Figure 3 illustrates this phenomenon.

**Figure 4:**
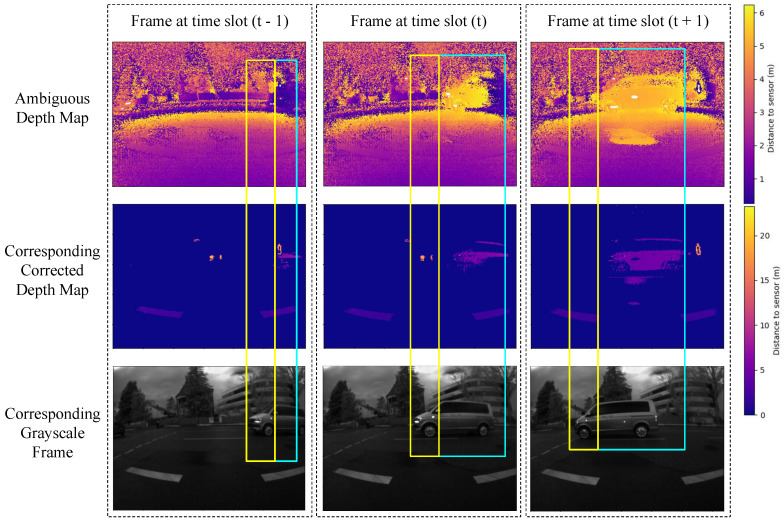
Samples from the dataset demonstrate the non-uniform spatial transformation of objects. The top row displays the ambiguous depth map, the middle row shows the corresponding ground truth depth map, and the bottom row depicts the corresponding grayscale images. The three columns represent three frames captured at subsequent time slots. Each blue box indicates that the car’s object in the ambiguous depth map is synchronized with the ground truth in the same frame. Meanwhile, the yellow box illustrates how the object’s position is shifted between the ambiguous depth map and the grayscale for the same frame.

Non-uniform temporal and spatial transformations complicate the prediction of the unwrapped depth map from the grayscale frame. Figure 4 provides examples of these transformations from the dataset.

## 3. Related Works

### 3.1. Motion Blur Noise Removal

The primary issue addressed in our method is motion blur. Gao et al. [15] studied motion blur in iToF sensors, categorizing it into half-frame (two out of four DCS) and full-frame blur (four out of four DCS), and proposed a mathematical model for correction. While their method effectively removes motion blur, it does not address the depth wrapping problem simultaneously. Their approach, using a 40 MHz modulation frequency, can remove blur for objects within a range of 1 m to 2 m, which corresponds to the maximum unambiguous range for this frequency, as per Equation (1).

Huo et al. [10] proposed a motion blur correction method using blind non-uniform motion deblurring with an Atrous Spatial Pyramid Deformable Convolution kernel. This kernel, based on residual inception [16], includes four modulation kernels with varying dilation rates and offset maps. Although their method is designed for compensating prolonged exposure time in traditional cameras, their concept inspired the development of our Atrous Spatial Inception Pyramid Module (ASIPM) and the Visual Attention Network, as detailed in Section 4.1.2.

Chang et al. [17] addressed motion blur and light saturation noise using a recurrent architecture designed for traditional cameras. However, this method is not suitable for iTOF sensors, as the motion blur noise in iTOF sensors differs from that in traditional cameras, as explained in Section 2.

Argaw et al. [18] and Zhang et al. [19] addressed motion blur in traditional cameras using optical flow estimation and the correction of low Signal-to-Noise Ratio (SNR) sharp images paired with high-SNR blurred, respectively. These methods rely on fusing a sharp low-SNR image with a blurred high-SNR image. Optical flow compensates for motion blur by adjusting for transformations in the opposite direction of the flow. However, this approach does not work for iTOF sensors, where the optical flow cannot compensate for motion blur due to the correlation of the four DCS frames creating the ambiguous depth map. Despite this, these methods influenced our approach, using grayscale as a sharp, low-SNR source and the ambiguous depth map as a high-SNR blurred source.

The presented methods are designed for 2D frames from traditional cameras, whereas our method focuses on 3D motion blur reduction using sharp grayscale images and ambiguous depth maps. We train the model on both static and dynamic datasets. While prior methods target 2D images, our solution employs an inverse blur block to address motion blur along the X and Y axes and resolves z-axis blur through phase unwrapping, effectively transforming the 3D blurring problem into a simpler 2D context.

### 3.2. Phase Unwrapping in Motion State

We address the challenge of phase unwrapping in iTOF Lidar systems, where ambiguity often obscures point cloud details across multiple cycles. While existing methods effectively unwrap ambiguous depth maps in static scenarios [20,21], they are unsuitable for motion states where motion blur introduces additional noise. Our proposed method specifically targets phase unwrapping under these dynamic conditions.

Wang et al. [22] used their VNet model, based on the UNet architecture, to unwrap ambiguous depth phases in Fringe Projection Profilometry (FPP), a Lidar technology different from AMCW iTOF Lidar. Similarly, Qiao et al. [23] proposed an architecture for noise removal and depth map unwrapping in static scenes. Schelling et al. [24] accurately predicted optical flow from iTOF Lidar during motion but limited their approach to the first cycle of unwrapped depth maps. All these methods are tailored for indoor scenes only.

Jung et al. [21] proposed the *Wild ToFu* method, which combines grayscale and DCS frames to produce unwrapped depth maps. However, it requires both inputs, making it unsuitable for night vision. Unlike traditional cameras, Lidar uses laser pulses to function in visually challenging environments such as smoke, dust, or darkness, where visible light fails [25]. Additionally, *Wild ToFu* is designed for static indoor scenes, while our method targets outdoor scenes, addressing challenges like varied lighting, dynamic elements, large-scale settings, and complex geometry [26].

Our method unwraps phases beyond the third cycle during motion while mitigating motion blur noise. It operates without requiring grayscale images, making it ideal for low-light scenarios such as night driving and tunnels. Furthermore, it can function with just two DCS frames, reducing stress on laser components.

### 3.3. Improving Model Predictions Using Continuous Learning

Our semi-supervised learning method begins by training the model on static scenes to leverage their sharpness, enabling it to predict unwrapped sharp depth maps from ambiguous ones. Subsequently, the model uses this learned ability to predict sharp depth maps from ambiguous, motion-blurred depth maps in dynamic scenes.

Continuous learning techniques support this approach by starting with a simple dataset and gradually progressing to more complex ones. While effective, continuous learning often encounters the challenge of catastrophic forgetting. Wang et al. [27] addressed this issue by training small parameter sets, known as prompts, to enhance continuous learning. Rehearsal-based continuous learning, which retains copies of old datasets, can be demanding. To overcome this, Smith et al. [28] proposed a rehearsal-free method using knowledge distillation and parameter regularization.

Our approach addresses catastrophic forgetting using knowledge distillation, combining multiple methods: the rehearsal technique, L2 regularization, and model regularization through dropout.

## 4. Depth Unwrapping with Motion Deblurring Method

Our proposed method addresses three simultaneous challenges: unwrapping depth map phases during motion, mitigating motion blur artifacts, and handling time shifts across frames. Given this complexity, we provide a formal background to lay the foundation for the design of our solution.

### 4.1. Method Formal Background

#### 4.1.1. Handling Grayscale Time Shift

The grayscale image is captured with a time difference from the ambiguity and ground truth depth maps. We utilize spherical coordinates to simplify the mathematical representations. This time shift induces non-uniform spatial transformations for grayscale pixels, approximated as an affine transformation matrix. The Spatial Transformer Network (STN) [29] addresses this issue by training the network through unsupervised learning to predict the inverse affine transformation matrix, thereby correcting pixel transformations. We represent the time shift in the grayscale image as follows:

(2) g=Af

where *f* represents the grayscale image without affine transformation, *g* is the time-shifted grayscale image captured by the sensor, and *A* is the affine matrix representing non-uniform transformation due to time shift. Equation (3a,b) show how the inverse matrix $A^{-1}$ is derived, ensuring that the final corrected grayscale image, denoted by $A^{-1}g$, is used as input for the depth unwrapping network.

(3a) $$\begin{array}{c} A^{-1}g=A^{-1}Af \end{array}$$

(3b) $$\begin{array}{c} A^{-1}g=f \end{array}$$

Our model utilizes the STN network to predict the inverse affine transformation matrix $A^{-1}$ used to correct the grayscale image time shift [29].

#### 4.1.2. Handling Ambiguous Depth Map Blurriness

Equation (4) defines motion blur as a convolution between a time-based sequence of blur kernels and the original image with additional noise [30,31].

(4) g=k∗f+n

Here, *f* represents the original image without motion blur, *k* is the unknown blur kernels tensor, *g* is the image with motion blur noise, and *n* is the additive Gaussian noise. Equation (5) defines the blur effect in the frequency domain.

(5) G=K·F+N

Here, *G* represents the motion-blurred image in the frequency domain, *K* denotes the motion blur kernel in the frequency domain, *F* is the sharp image in the frequency domain, and *N* is additive Gaussian noise in the frequency domain. Balancing the removal of blur and the preservation of image details is important when designing anti-blurring filters. Gaussian noise typically manifests as high-frequency variations in pixel values, and anti-blurring filters often aim to enhance high-frequency components while attenuating low-frequency components associated with blur. Since it is not required to keep Gaussian noise, then we can simplify Equation (5) by neglecting Gaussian noise; hence, we can use Equation (6) [31].

(6) G=K·F

Using blind deconvolution [31], we try to find the term $K^{-1}$, representing all optical flow transformation kernels per pixel to reverse the blur effect according to Equation (7).

(7) $$K^{-1}\cdot G=F$$

Mathematically, it is challenging to calculate $K^{-1}$ due to the non-invertible nature of the convolution operator. We propose a deep learning model that uses Equation (8) to estimate h(k) such that it minimizes the difference between the predicted sharp image s(f) and the actual sharp image *f*.

(8) h(k)∗g=s(f)

Equation (9) presents the optimization problem, where θ is the deep learning model predicting the final depth image.

(9) θ(s(f))−gt≈0

Equation (10) illustrates how the model predicts the final depth map from the motion-blurred image, using an unsupervised approach to predict the inverse blur kernel h(k), where gt is the ground truth image.

(10) θ(h(k)∗g)−gt≈0

**Figure 5:**
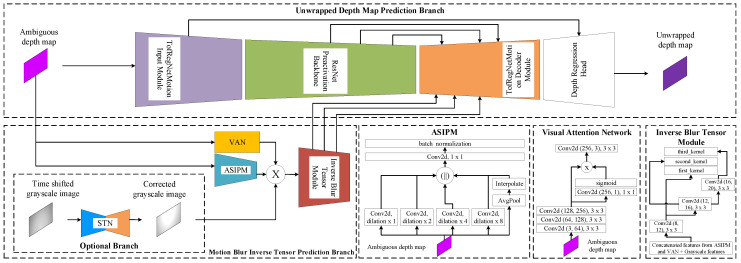
The model architecture comprises two branches. The first branch is the Main Unwrapped Depth Map Prediction Branch, an autoencoder architecture with four stages: the TofRegNetMotion input feature extraction stage for extracting overall depth context; ResNet Preactivation backbone for detailed feature extraction; TofRegNetMotion output decoder backbone for simultaneous prediction of unwrapped depth map and removal of motion blur; and depth regression head for the final detailed prediction of unwrapped depth map. The other branch is the Motion Blur Inverse Tensor Prediction Branch. This consists of three primary components: The ASIPM and Visual Attention Network modules process the wrapped depth map simultaneously, and the Inverse Blur Tensor module generates the final motion blur inverse tensor feature map. An additional option branch for the grayscale synchronization branch based on the STN network may be used in concatenation with the ASIPM and Visual Attention Network.

Our ASIPM, the Visual Attention Network, and the inverse blur tensor modules represent the model θ used to predict the inverse motion blur tensor. As illustrated in Figure 5, the ASIPM module operates on the ambiguous depth map; it aims to extract features about the optical flow of vertices of the point cloud in a general frame context. On the other hand, the visual attention network takes the ambiguous depth map as input. It implements a simple visual attention mechanism for the input to extract the optical flow of point cloud vertices on the object’s scope. The inverse blur tensor module inputs the concatenation of the output of the visual attention and ASIPM modules. It predicts the tensor of three kernels representing the inverse optical flow features for the point cloud vertices.

#### 4.1.3. Phase Unwrapping in the Dynamic State

Current depth correction techniques for AMCW sensors primarily address static datasets [32] (i.e., where the sensor and the scene’s contents are not moving at all), overlooking dynamic scenarios (i.e., where the sensor and the scene’s contents are moving) where motion blur impacts depth maps. State-of-the-art models can accurately predict sharp depth maps in static scenarios, such as when a vehicle is parked inside a garage, where there is no motion for the vehicle or the other objects in the scene.

We hypothesize that if the model is trained to accurately predict sharp unwrapped depth maps from static scenes with no motion blur, then using continuous learning, we can build on this skill to make it able to predict unwrapped depth maps with reduced motion blur from dynamic scenes.

Our proposed model’s prediction accuracy can be represented by multivariate probability density functions that define the relationship between the input ambiguous depth map and the ground truth unwrapped depth map. To achieve accurate prediction, we aim to make the likelihood of predicting a sharp, unwrapped depth map from a dynamic scene similar to predicting a sharp, unwrapped depth map from a static scene. Let the probability density function $P_{s}\left(Y_{s},X_{s}\right)$ represent prediction accuracy in the static dataset $D_{s}$ by capturing the correspondence between the static input $X_{s}$ representing the static scene ambiguous depth map and the corresponding prediction space $Y_{s}$ representing the ground truth sharp unwrapped depth map. Similarly, $P_{d}\left(Y_{d},X_{d}\right)$ represents prediction accuracy in the dynamic dataset $D_{d}$ by capturing the relationship between the dynamic input $X_{d}$ representing the dynamic scene ambiguous depth map and the dynamic prediction space $Y_{d}$ representing the ground truth sharp unwrapped depth map. We can express the model’s prediction in a static state using Bayes’ theorem:

(11) $$P_{s}\left(X_{s}|Y_{s}\right)=\frac{P_{s}\left(X_{s},Y_{s}\right)}{P_{s}\left(Y_{s}\right)}$$

where $P_{s}\left(X_{s},Y_{s}\right)$ is the joint probability of predicting the ground truth static depth map $Y_{s}$, and using the static input ambiguous depth map $X_{s}$, $P_{s}\left(X_{s}|Y_{s}\right)$ is the likelihood that the static input $X_{s}$ would result in predicting the ground truth static depth map $Y_{s}$, and $P_{s}\left(Y_{s}\right)$ is the marginal accuracy of the model to predict the static ground truth depth map $Y_{s}$, regardless of which input $X_{s}$ is used. Similarly, the model’s prediction in a dynamic state can be expressed as follows:

(12) $$P_{d}\left(X_{d}|Y_{d}\right)=\frac{P_{d}\left(X_{d},Y_{d}\right)}{P_{d}\left(Y_{d}\right)}$$

$P_{d}\left(X_{d},Y_{d}\right)$ is the joint probability of predicting the ground truth dynamic depth map $Y_{d}$, and using the dynamic input ambiguous depth map $X_{d}$, $P_{d}\left(X_{d}|Y_{d}\right)$ is the likelihood that the dynamic input $X_{d}$ would result in predicting the ground truth dynamic depth map $Y_{d}$, and $P_{d}\left(Y_{d}\right)$ is the marginal accuracy of the model to predict the ground truth dynamic depth map $Y_{d}$, regardless of which input $X_{d}$ is used.

If the prediction accuracy of the probability density function in the dynamic dataset closely resembles that of the static dataset, and assuming the accuracy of predicted ground truth sharp unwrapped static depth maps $Y_{s}$ is similar to that of predicted ground truth sharp unwrapped dynamic depth maps $Y_{d}$, then the model employed for precise predictions in the static dataset could also be effective for the dynamic dataset. This assumption is bold since having two statistical models dependent on different input variables but can give similar prediction accuracy is challenging. However, this assumption holds when the dynamic state model is trained with random batches from the static dataset and the vehicle moves at low speeds, minimizing time shifts within the range of **0** km/h to **30** km/h, as typically seen in parking scenarios. This technique ensures that a similarity condition is employed for the two probability density functions. This condition is essential for the assumption to hold [33]. Equation (13a–e) show our mathematical induction that inputs from the dynamic dataset can be used to predict sharp unwrapped depth maps with an accuracy approximately equivalent to that of the static dataset.

(13a) $$\begin{array}{c} :P_{s}\left(Y_{s},X_{s}\right)\approx P_{d}\left(Y_{d},X_{d}\right) \end{array}$$

(13b) $$\begin{array}{c} :P_{s}\left(Y_{s}\right)≃P_{s}\left(Y_{d}\right) \end{array}$$

(13c) $$\begin{array}{c} ∵P_{s}\left(X_{s}|Y_{s}\right)=\frac{P_{s}\left(Y_{s},X_{s}\right)}{P_{s}\left(Y_{s}\right)} \end{array}$$

(13d) $$\begin{array}{c} ∵P_{d}\left(X_{d}|Y_{d}\right)=\frac{P_{d}\left(Y_{d},X_{d}\right)}{P_{d}\left(Y_{d}\right)} \end{array}$$

(13e) $$\begin{array}{c} ∴P_{s}\left(X_{s}|Y_{s}\right)\cdot P_{s}\left(Y_{s}\right)\approx P_{d}\left(X_{d}|Y_{d}\right)\cdot P_{d}\left(Y_{d}\right) \end{array}$$

We aim to achieve equivalence between the likelihood of accurate prediction for the dynamic depth map ${\hat{Y}}_{d}$ from the input $X_{d}$ and accurate prediction for the static depth map ${\hat{Y}}_{s}$ from the input $X_{s}$:

(14) $$P_{s}\left(X_{s}|{\hat{Y}}_{s}\right)≃P_{d}\left(X_{d}|{\hat{Y}}_{d}\right)$$

Our hypothesis, as described in Section 4.1.3, is that this assumption can hold if we can make the continuous learning able to equalize the likelihood of accurately predicting ${\hat{Y}}_{d}$ from input $X_{d}$ with that of accurately predicting ${\hat{Y}}_{s}$ from input $X_{s}$. This equilibrium can be realized if the static deep learning model $\theta_{s}$ can produce similar predictions to the dynamic deep learning model $\theta_{d}$. The model $\theta_{d}$ should be trained using the dataset $D_{d}+f\left(D_{s}\right)$ to achieve this equilibrium, where $f(D_{s})$ represents the function correlating the original static dataset $D_{s}$ to the dynamic dataset $D_{d}$. Notably, $f\left(D_{s}\right)\subseteq D_{s}$ ensures that learning from the dynamic dataset does not cause catastrophic forgetting of the static dataset; refer to Section 3.3 to understand the approach.

### 4.2. Method Implementation Details

The proposed solution integrates a continuous learning framework for adapting from static to dynamic datasets and a computer vision model for depth unwrapping in dynamic scenes, employing techniques outlined in Section 4.1.

#### 4.2.1. Proposed Model Architecture

The architecture of the model we propose here is based on the autoencoder architecture as illustrated in Figure 5.

The model comprises two branches:

- **Main Branch, Unwrapped Depth Map Prediction Branch:** an autoencoder architecture with four stages:
  - The **TofRegNetMotion input encoder** is the first input stage. It is designed to deal with ambiguous depth map details to extract the overall depth context. It comprises four subsequent vanilla ResNet blocks [34].
  - The **ResNet Preactivation backbone** is the following stage, designed for detailed depth feature extraction. The stage is composed of eight subsequent Preactivation ResNet blocks [35].
  - The **TofRegNetMotion output decoder** is designed to predict unwrapped depth maps and reduce motion blur simultaneously. It fuses the feature block extracted with the ResNet Preactivation backbone and the features extracted by the motion blur inverse tensor prediction branch to perform the prediction task. The module is composed of four subsequent up-sampling residual blocks.
  - The **depth regression head** predicts the final detailed unwrapped depth map with reduced motion blur. It uses the output of the TofRegNetMotion input encoder stage and the TofRegNetMotion output decoder stage through skip connections. The module comprises a transposed convolution layer followed by two convolution layers.
- **Motion Blur Inverse Tensor Prediction Branch:** Consists of three primary components, as in Figure 5:
  - The **ASIPM module** processes the wrapped depth map to predict the general context optical flow of the point cloud vertices. It comprises four different CNN kernels, based on residual inception [16], with four different dilation levels (one, two, four, and eight dilation kernels) followed by a feature fusion CNN kernel composed of an adaptive average pooling layer followed by two convolution layers, a batch normalization layer, and a ReLU activation layer.
  - The **Visual Attention Network module** processes the wrapped depth map simultaneously. It implements a simple visual attention mechanism [36,37] for the input. The module comprises three subsequent convolution layers followed by a self-attention layer.
  - The **Inverse Blur Tensor module** generates the final motion blur inverse tensor feature map. It concatenates the output of the visual attention and ASIPM modules. Then, it applies cascaded convolution kernels to estimate the final inverse blur tensor. The module comprises a concatenation layer followed by three dilation convolution kernels, each of which has a dilation size of two.

The output tensor of the inverse blur tensor module serves as an additional feature map for the TofRegNetMotion output decoder stage in the main branch.

Furthermore, an alternative architecture with an additional branch may enhance the accuracy of predicted motion blur inverse tensors by utilizing the grayscale image as a sharp, low-SNR depth map synchronized with the depth-wrapped depth map through an STN network [29]. If activated in our proposed model, the output of the STN will be concatenated with the output of the ASIPM and Visual Attention Network modules. Refer to the ablation study in Section 5.4 and Table 3 to observe the impact of removing this branch from the model.

#### 4.2.2. The Continuous Learning Framework

**Figure 6:**
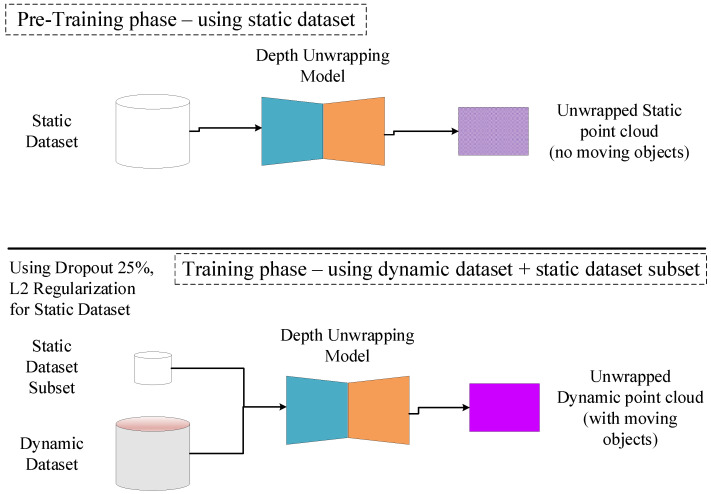
**Continuous training framework.** The model is trained only using the static dataset during the pretraining phase. During the training phase, the model is trained using the dynamic dataset and a subset of the static dataset, represented by random batches.

As Figure 6 illustrates, the continuous learning mechanism comprises two phases. Initially, the model is trained on the static dataset to predict static unwrapped sharp depth maps. Following this, it undergoes training on the dynamic dataset to predict such maps under motion. To address catastrophic forgetting, three methods are employed.

- Dropout layers are integrated into the model, activated solely during training with the dynamic dataset. These layers aim to prevent overfitting to the dynamic dataset, thereby retaining knowledge about the static dataset and promoting generalization [38].
- The rehearsal technique involves randomly selecting batches from the static dataset during training with the dynamic dataset. This selection process ensures representation from the entire static dataset, preventing overfitting to specific scenarios. By incorporating batches from the static dataset, the model retains previous knowledge while learning from the dynamic dataset [39].
- L2 regularization is applied by amplifying the loss function for static dataset batches during training with the dynamic dataset, effectively penalizing inaccurate predictions [40]. When training the model with the dynamic dataset, the loss function for static dataset batches is multiplied by a factor greater than 1.0. This over-penalization ensures that the model prioritizes fitting to the static dataset, thereby retaining its knowledge even during training with dynamic data [40].

Although the continuous learning technique proves to be effective, it could be computationally expensive and time-consuming. This drawback can be solved thanks to vehicle connectivity, modern cloud computing platforms, and advanced parallel computing architectures like Tensor Processing Units (TPUs) from Google and Tensor Core GPUs from Nvidia; these architectures are designed to serve intensive AI training workloads at acceptable power consumption requirements, while also achieving highly satisfactory computational performance and acceptable execution time frames.

#### 4.2.3. Loss Functions

The loss functions used for each training phase are a key component for training the model. The loss functions are mainly composed of four components. The first component is the Huber loss [24] function,

(15) $$L_{\delta }\left(y,\hat{y}\right)=\left\{\begin{array}{c} \frac{1}{2}{\left(y-\hat{y}\right)}^{2},\mathrm{if}\;\left|y-\hat{y}\right|\leq \delta ,\\ \delta (|y-\hat{y}|-\frac{1}{2}\delta ),\mathrm{otherwise}. \end{array}\right.$$

This function is used as the mainstream for penalizing the model; it is very suitable for handling outliers and smooth depth. The other major component is the structural similarity index measure loss [41],

(16) $$L_{\mathrm{SSIM}}\left(y,\hat{y}\right)=\frac{\left(2\mu_{y}\mu_{\hat{y}}+C_{1}\right)\left(2\sigma_{y\hat{y}}+C_{2}\right)}{\left(\mu_{\hat{y}}^{2}+\mu_{y}^{2}+C_{1}\right)\left(\sigma_{\hat{y}}^{2}+\sigma_{y}^{2}+C_{2}\right)}$$

where $C_{1}$ and $C_{2}$ are selected empirically to be $0.01^{2}$ and $0.03^{2}$, respectively. In addition to this, we apply our depth-guided loss function, which is defined as follows:

(17) $$L_{\mathrm{dg}}\left(y,\hat{y}\right)=\alpha L_{\mathrm{top}}\left(y,\hat{y}\right)+\beta L_{\mathrm{gradient}}\left(y,\hat{y}\right)+\gamma L_{\mathrm{smooth}}\left(\hat{y}\right)$$

where α, β, and γ are selected empirically to be 0.2, 0.7, and 0.1, respectively. The $L_{\mathrm{top}}$ loss function is defined as,

(18) $$L_{\mathrm{top}}\left(y,\hat{y}\right)=MSE\left(top\_max\left(y,\gamma \right),top\_max\left(\hat{y},\gamma \right)\right)$$

where γ defines the number of top maximum elements for the input and is empirically selected to be 1200. The $L_{\mathrm{gradient}}\left(y,\hat{y}\right)$ loss function is defined as,

(19) $$L_{\mathrm{gradient}}\left(y,\hat{y}\right)=\frac{1}{N}\sum_{i=1}^{N}{∥\nabla y_{i}-\nabla {\hat{y}}_{i}∥}_{2}^{2}$$

and the $L_{\mathrm{smooth}}\left(\hat{y}\right)$ loss function is defined as,

(20) $$L_{\mathrm{smooth}}\left(\hat{y}\right)=\frac{1}{N}\sum_{i=1}^{N}∥\nabla {\hat{y_{y}}}_{i}+\nabla {\hat{y_{x}}}_{i}∥$$

This depth-guided loss function removes depth noise caused by significant changes in the gradient. It also ensures that large continuous depth segments are homogeneous point clouds. In addition, it tackles a common problem in computer vision: the accuracy of depth prediction decreases dramatically as the object’s depth increases.

The last component of the loss function is the scale-invariant loss,

(21) $$\begin{array}{c} L_{\mathrm{siv}}\left(\hat{y},y\right)=\frac{1}{2N}\sum_{i=1}^{N}{\left(\log{\hat{y}}_{i}-\log y_{i}\right)}^{2}\\ -\frac{1}{2N^{2}}{\left(\sum_{i=1}^{N}\log{\hat{y}}_{i}-\log y_{i}\right)}^{2} \end{array}$$

The scale-invariant loss addresses depth saturation scenarios where depth regions become undefined. It ensures that in the presence of depth discontinuities, the model can estimate depth based on object shapes and surroundings, regardless of the object’s absolute depth, which is unavailable in saturation cases.

The overall loss function is composed of the previously stated components, as follows:

(22) $$L=aL_{\delta }+bL_{\mathrm{dg}}+cL_{\mathrm{siv}}+dL_{\mathrm{SSIM}}$$

where weights *a*, *b*, *c*, and *d* are selected empirically to be 40.0, 45.0, 25.0, and 40.0, respectively. These values are selected to be much greater than 1.0 to avoid the vanishing gradient problem.

The same loss function trains the static and dynamic datasets phases. However, during the dynamic dataset phase, to apply L2 regularization, when the batches selected from the static dataset are used to train the model, the overall loss function is multiplied by a factor:

(23) $$L_{static}=\rho L_{dynamic}$$

where ρ is empirically selected to be 1.2.

#### 4.2.4. Learning Rate Decay Scheduling

Training a complex model requires careful hyperparameter selection, with the learning rate being crucial. Constant rates lead to quick convergence to local minima; techniques like learning rate decay scheduling solve this issue. Kaichao et al. [42] discuss scheduling algorithms, showing the efficacy of learning rate decay in transfer tasks. Similarly, Jinia et al. [43] explore scheduling methods. Our experiments identify step decay as optimal, starting with a high learning rate and gradually reducing it.

## 5. Experiments and Results

We used the Valeo NFL sensor [6], provided by Valeo Schalter und Sensoren GmbH, Bietigheim-Bissingen, Germany which uses the ESPROS imager [8], provided by ESPROS Photonics AG, Sargans, Switzerland. Our experiments were based on our internally recorded datasets for static and dynamic scenarios.

### 5.1. Training Details

Both the pretraining and training phases utilized the Adam optimizer with default values. The learning rate followed a stepwise decay algorithm, starting at 0.0007 and reaching a minimum of 0.0003 for both phases. The static dataset underwent training for 30 epochs with a decay rate every six steps, while the dynamic dataset was trained for 30 epochs with a decay rate every ten steps. A 25% dropout rate was applied during the dynamic dataset training phase. Both training phases used a batch size of 16. The model was implemented using PyTorch version 2.2.0 LTS and trained using an Nvidia RTX 3060 GPU from Nvidia, purchased from Stuttgart, Germany.

### 5.2. Datasets

Two datasets were collected. The static dataset included scenarios where scenes were captured while the vehicle and environment were not in motion. Conversely, the dynamic dataset involved scenarios for parking out, parking in, and driving in low-speed areas, where both the vehicle and environmental objects were in motion.

**Figure 7:**
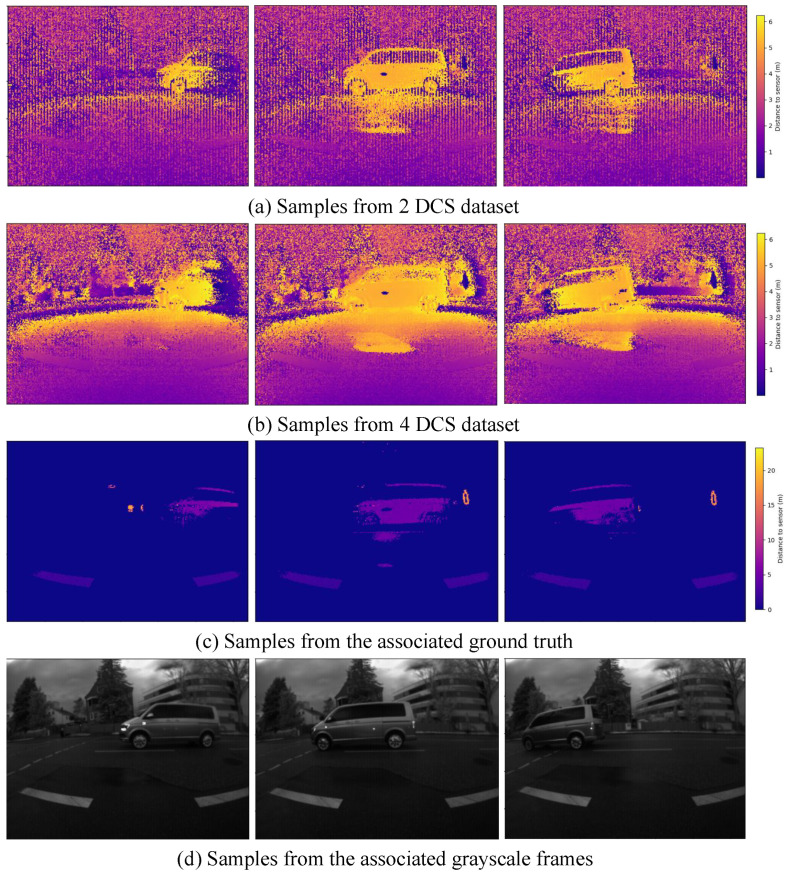
**Samples from the 4-DCS dataset and 2-DCS dataset.** Images in row (**a**) show ambiguous depth maps created using 2-DCS frames; images in row (**b**) show the corresponding ambiguous depth created using 4-DCS frames; images in row (**c**) show corresponding ground depth maps with correct depth; and images at row (**d**) show corresponding grayscale frames.

The NFL sensor generated unwrapped depth maps by applying the dual-frequency modulation method [11]. These maps were used as ground truth maps. The resulting ground truth map was then recorded alongside the associated DCS and grayscale frames to create the dataset samples. Figure 7 shows samples of both datasets.

We defined our dataset samples by the keyframe. A keyframe is a distinct, non-repeated frame composed of an ambiguous depth map, a corresponding ground truth unwrapped depth map, a corresponding grayscale frame, and a corresponding laser amplitude frame. According to the sensor’s recording sequence, these components were recorded from the same time reference point but in different time shifts. Data augmentation was applied to the training keyframes. Each keyframe was flipped horizontally, vertically, and rotated 180° around the z-axis, generating four different frames, the original one and three other augmented frames. The static dataset contained 1 K keyframes for training, 120 keyframes for validation, and 120 keyframes for testing. The dynamic dataset contained 3.3 K keyframes for training, 430 keyframes for validation, and 430 keyframes for testing. The datasets were captured using both two-DCS frames and four-DCS frames per modulation frequency.

### 5.3. Results

#### 5.3.1. Comparative Analysis

As we illustrated in our literature review, our proposed method is the first one designed to tackle the problems of ambiguous depth map unwrapping during motion and reducing the motion blur simultaneously. We showed in Section 3 that the ambiguous depth measurement and motion blur trailing traces are two tightly coupled problems that impact each other. A solution that tackles the depth ambiguity problem during motion should also consider motion blur removal to improve depth accuracy.

So far, only two methods have made partial progress in addressing these challenges. The first, developed by Gao et al. [15], focuses on motion blur reduction but does not provide a solution for depth unwrapping. It can only operate within the unambiguous range. The second, developed by Jung et al. [21], known as Wild ToFu, does offer a solution to unwrap ambiguous depth maps, but it is limited to static indoor scenes. This analysis explains why we cannot compare our work to the existing state-of-the-art.

However, we tried to benchmark our proposed model results using the static dataset versus Wild ToFu results to evaluate the model’s performance against the state-of-the-art even for the static scenes. Considering that Wild ToFu is designed for indoor scenes with a different sensor, our method is designed for outdoor scenes based on the Valeo NFL sensor; we had to make some adaptations to the model of Wild ToFu to be compatible with our static dataset labels. The Wild ToFu method works directly on the four DCS frames. Also, the sensor’s frame size used with Wild ToFu is 640×480. On the other hand, our method operates directly on the ambiguous depth map of frame size 320×240. So, we have adapted the input layer of Wild ToFu to be compatible with our labels.

Despite the challenges posed by the outdoor environment, such as longer measured distances, sunlight interference, and environmental factors, our method has proven its adaptability and superiority. Table 1 provides a comprehensive comparison between the adapted Wild ToFu method and our proposed method, trained on the static dataset of our sensor. The results demonstrate that our method outperforms the adapted Wild ToFu; note that GS refers to the grayscale frame.

On the other hand, for motion blur reduction, as illustrated previously, no prior state-of-the-art method has been proposed to solve the problem while unwrapping the ambiguous depth; hence, we cannot provide any comparative analysis for this part, and our model acts as a baseline for further developments in this space.

#### 5.3.2. Quantitative Analysis

We start first with the quantitative results of the model. Table 2 summarizes the performance of our method when it is used in dynamic scenarios, in addition to the common prediction error measurements for the depth estimation function. Upon initial analysis, it becomes apparent that the model’s accuracy is lower in dynamic scenarios compared to static scenarios. However, it is essential to acknowledge that ground truth data in dynamic scenarios may be affected by motion blur, potentially influencing accuracy assessments. To ensure a fair evaluation, the model’s performance is primarily assessed in static scenarios where motion blur is absent. The model’s strong performance in static scenarios, with the absence of motion blur, indicates its adaptability and potential to address this challenge, enabling better performance in dynamic scenarios.

The model’s proficiency in static scenarios, characterized by the absence of motion blur, provides insights into its behavior during low-speed driving in dynamic scenarios. Results in Table 1 demonstrate high accuracy in static scenarios, suggesting the potential for even better performance in dynamic situations with reduced motion blur. This outlook supports the hypothesis that the model’s accuracy is influenced by its ability to handle motion blur effectively.

Additionally, factors such as depth filters applied in ground truth and ambiguous depth maps, irregularities like blooming, and multi-path interference impact the captured level of detail. The prediction model captures more information than the ground truth, leading to anomalies in the RMSE (Root Mean Square Error) metric calculation. A qualitative analysis will further explore the impact of these factors on the model’s performance. The reasons for the model’s low accuracy in dynamic scenarios can be summarized as follows:

1. The ground truth is affected by motion blur, while predicted maps address this issue, resulting in a higher error rate between the prediction and the ground truth.
2. The model’s predictions may capture more details than those detected by the ground truth, contributing to error calculation discrepancies.
3. External factors like blooming and sunlight interference impact both ground truth capture and prediction, although this falls beyond the scope of this paper.

In the Section 5.3.3, we delve into these findings and hypotheses. Despite the initial interpretation suggesting modest model performance, a deeper investigation reveals that our model surpasses the state-of-the-art method using dual modulation frequency in dynamic scenarios.

#### 5.3.3. Qualitative Analysis

**Figure 8:**
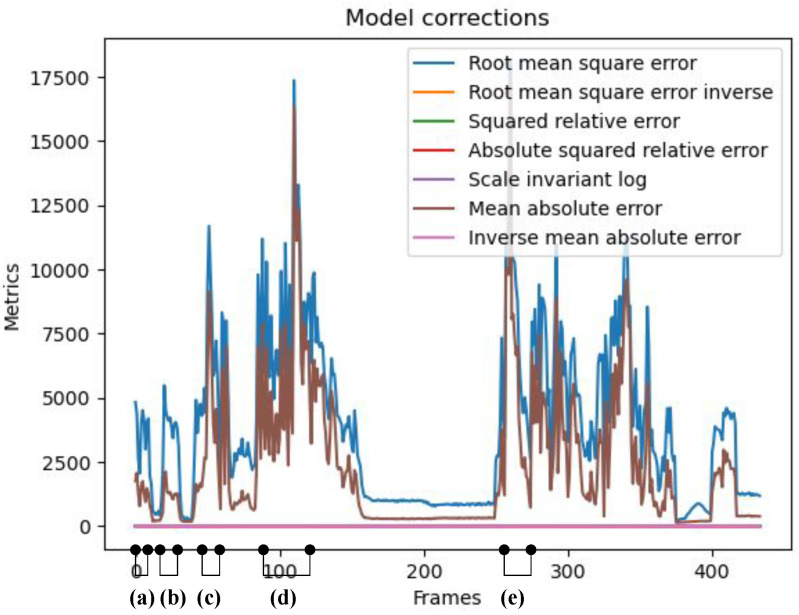
The analysis focuses on the test frames of the 2-DCS dataset, with selected frame groups based on RMSE and MAE values. The targeted groups comprise frames with high RMSE and MAE values. As per the selection criteria, frames are categorized into groups: (a) frames 0 to 6; (b) frames 8 to 18; (c) frames 56 to 62; (d) frames 90 to 115; and (e) frames 250 to 260. The most significant metric graphs in the figure are the RMSE and MAE graphs; the other metric graphs (i.e., iRMSE, iMAE, SqRel, AbsSqRel, and SiL) are of very small value, and so they are superimposed over each other near a value of 0.

We initiate our qualitative analysis by identifying frames with the highest RMSE and MAE (Mean Absolute Error). Figure 8 illustrates the selected frames for further examination. The figure describes a trace of 500 test frames captured by the sensor and corrected using our method. The graphs in the frame show the RMSE and MAE between the corrected frames and the ground truth. In the figure, we have identified five different groups of frames (i.e., frames groups a, b, c, d, and e), and each group shares a common reason for high RMSE and MAE values. The qualitative analysis focused on analyzing these reasons and providing thorough explanations and justifications. The significance of our findings lies in the potential to improve sensor technology. Due to space constraints, we will present a subset of samples from each group for review.

It is important to note that the primary reason for the high RMSE and MAE values observed between the corrected and ground truth frames stems from using an iToF Lidar sensor to capture the ground truth frames that implement a depth cutoff filter for point cloud depth values exceeding 25 m. Our method does not utilize this depth filter, allowing for the detection of objects beyond 25 m. Also, the ground truth is susceptible to errors. It does not perfectly reflect “reality” due to motion blur since there is no state-of-the-art solution for this problem in iTOF Lidar sensors, even though it offers accurate unwrapped depth maps. Therefore, the recreated point cloud by our proposed model might exhibit a closer resemblance to reality than the ground truth despite displaying “inaccurate” results. Additionally, the resolution of the CMOS imager in the NFL sensor used for predictions and ground truth is 320×240 pixels, which is relatively low compared to state-of-the-art RGB and grayscale camera frames. This limitation helps explain the low resolution of the provided frames. We plan to tackle these ground truth issues in future work, as will be discussed in Section 6.

**Figure 9:**
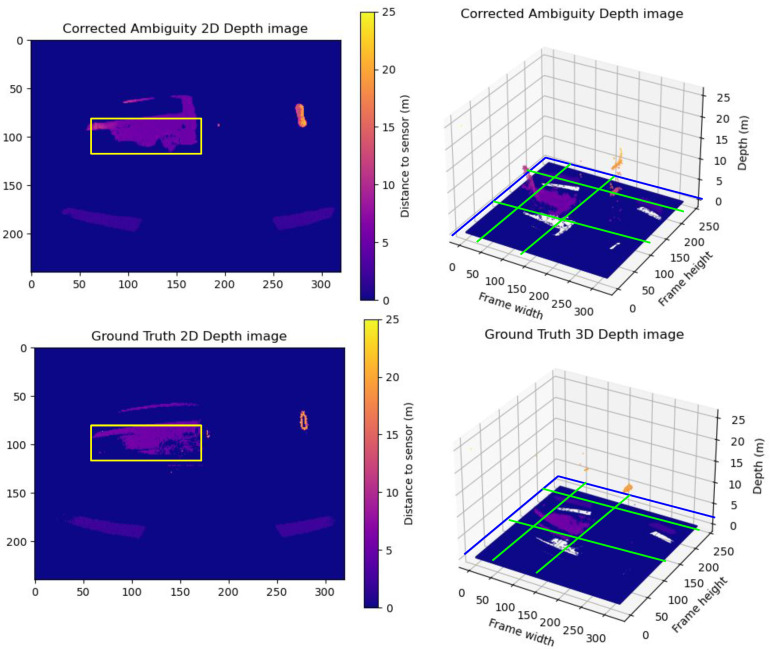
A sample from Group A frames. The upper row shows the predicted unwrapped depth maps in 2D and 3D views. The lower row shows the ground truth unwrapped depth maps in 2D and 3D views. As shown in the figure, the vehicle point cloud highlighted inside the yellow box in the corrected depth map in the upper row has less motion blur trailing traces and more homogeneous sharp depth than the corresponding vehicle point cloud in the ground truth in the lower row, while maintaining the same level of detail for the point cloud of the scene. The yellow boxes in the 2D frames compare the correction results between the corrected depth and ground truth frames. The Green lines show the cross-pending corrected areas in the point cloud in the 3D point cloud.

**Group A Frames:** This group exemplifies the impact of motion blur on depth prediction accuracy. Despite closely matching point clouds between the corrected frames and the ground truth, the corrected frames exhibit more uniform point clouds. A sample of Group **A** is illustrated in Figure 9. The vehicle’s outer frame and the wheels are more rigid and defined—this effect of homogeneity in the corrected point cloud results in high differences in the RMSE and MAE.

**Figure 10:**
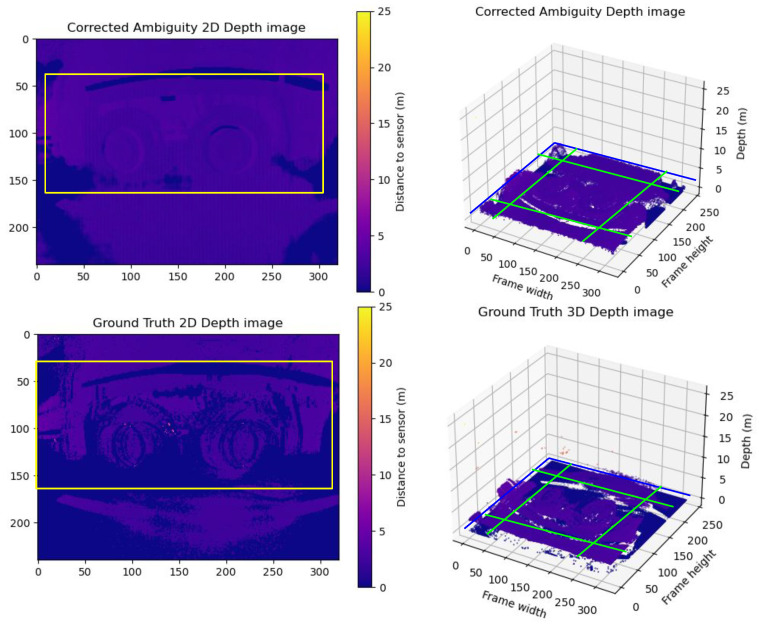
A sample from Group B frames. The upper row shows the predicted unwrapped depth maps in 2D and 3D views. The lower row shows the ground truth unwrapped depth maps in 2D and 3D views. As shown in the figure, the ground truth point cloud suffers badly from motion blur trailing traces to the extent that there are several shadows for the wheels. On the other hand, the model could reduce the motion blur trailing traces and sharpen the details of the objects like the wheels. The yellow boxes in the 2D frames compare the correction results between the corrected depth and ground truth frames. The Green lines show the cross-pending corrected areas in the point cloud in the 3D point cloud.

**Group B Frames:** Here, extensive blurriness noise corrupts the ground truth’s point cloud. The model partially smooths the depth, resulting in a more uniform point cloud with acceptable pixel gradient transitions, leading to differences between the ground truth and the prediction. A sample of Group **B** is illustrated in Figure 10. The wheels in the real world are of homogeneous depth; it is clear that the ground truth is very noisy, with very high gradients between almost every two neighboring pixels. The model was able to smooth the depth to some extent, which resulted in a homogeneous point cloud with acceptable gradients between neighbor pixels, but the details are not very sharp. This kind of noise is expected due to the scale of the blurred area.

**Figure 11:**
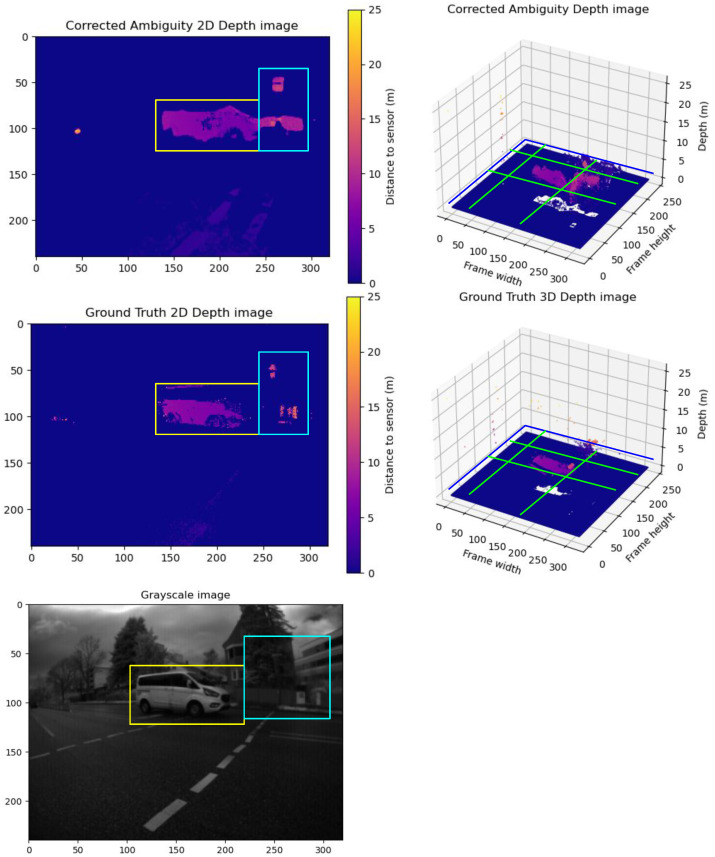
A sample from Group C frames. The upper row shows the predicted unwrapped depth maps in 2D and 3D views. The lower row shows the ground truth unwrapped depth maps in 2D and 3D views. In addition, we show the grayscale frame capture along with the ground truth. The vehicle point cloud inside the yellow box in the corrected depth map shows a sharper point cloud with reduced motion blur and reduced trailing traces compared to the vehicle point cloud in the ground truth. The blue box shows the captured detailed comparison between the corrected depth map and the ground truth map to the grayscale frame. It is evident that the corrected depth map contains more details than the ground truth, and these details are not just noise or prediction error, but they represent valid details compared to the grayscale frame. The yellow boxes in the 2D frames compare the correction results between the corrected depth and ground truth frames. The Green lines show the cross-pending corrected areas in the point cloud in the 3D point cloud.

**Group C Frames:** This sample showcases elevated RMSE and MAE due to the model’s enhanced performance. The model not only corrects blurriness but also extracts additional details from ambiguous depth maps beyond the ground truth. A sample of Group **C** is illustrated in Figure 11. The model not only corrected the blurriness but also extracted more details from the ambiguous depth maps compared to the ground truth. Here, we show the grayscale to show that extracted details are not just noise due to the model’s poor performance but are real objects. This is mainly due to the depth filter implemented in the sensor for capturing the ground truth point cloud frames.

**Figure 12:**
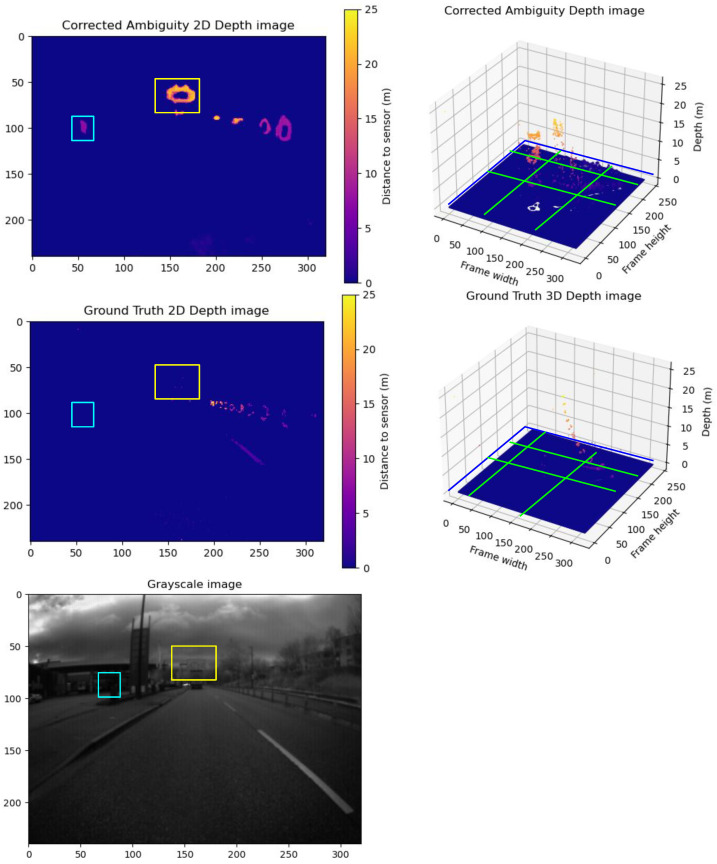
A sample from Group D frames. The upper row shows the predicted unwrapped depth maps in 2D view and 3D view. The lower row shows the ground truth unwrapped depth maps in 2D view and 3D view. In addition, we show the grayscale frame capture along with the ground truth. The blue and yellow boxes in the corrected depth maps show captured details not detected by the ground truth. Compared to the grayscale, it is not just a prediction error; they are accurate details not captured in the ground truth. The yellow boxes in the 2D frames compare the correction results between the corrected depth and ground truth frames. The Green lines show the cross-pending corrected areas in the point cloud in the 3D point cloud.

**Group D Frames:** Here, the model surpasses the ground truth by identifying more objects than are visible in the ground truth. This over-performance results in higher RMSE and MAE values. A sample of Group **D** is illustrated in Figure 12. The model was able to extract more objects than what is visible by the ground truth. The grayscale image shows that these are real objects and not just noise. This over-performance leads to higher RMSE and MAE values.

**Figure 13:**
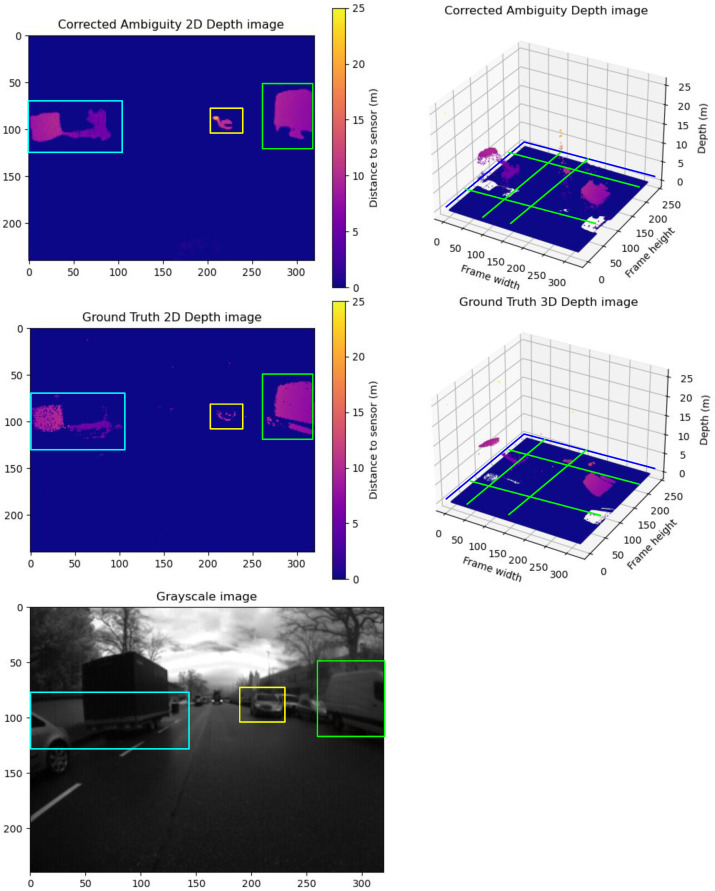
A sample from Group E frames. The upper row shows the predicted unwrapped depth maps in 2D and 3D views. The lower row shows the ground truth unwrapped depth maps in 2D and 3D views. In addition, we show the grayscale frame capture along with the ground truth. Comparing the point clouds of the objects inside the blue, yellow, and green boxes in the corrected and ground truth depth map, the corrected depth map has more details and a sharper point cloud. Comparing the corrected depth map to the grayscale, it is evident that it captures more valid details with less motion blur noise than the ground truth. The yellow boxes in the 2D frames compare the correction results between the corrected depth and ground truth frames. The Green lines show the cross-pending corrected areas in the point cloud in the 3D point cloud.

**Group E Frames:** Similar to Group D, the model exceeds the ground truth by identifying additional objects and generating a more uniform point cloud by filling holes in the depth segment. This over-performance leads to higher RMSE and MAE values. A sample of Group **E** is illustrated in Figure 13. The model generated a homogeneous point cloud where the holes in the depth segment were removed. The grayscale image shows that these are real objects and not just noise. This over-performance leads to higher RMSE and MAE values.

An interesting observation is the higher accuracy and quality of results obtained using two-DCS compared to four-DCS. This can be attributed to the fact that two-DCS ambiguous depth maps experience less motion blur since they are composed of fewer superimposed DCS frames. Consequently, two-DCS ambiguous depth maps are generally less noisy and suffer from fewer multi-path interferences.

### 5.4. Ablation Study

This ablation study was designed to ensure that each component in the framework contributed to the model’s overall performance. Two main activities were undertaken to conduct the study. Firstly, the importance of each component of the modules was examined. Secondly, the significance of continuous learning was evaluated.

To verify the hypothesis stated in Section 4.1.3, an ablation study was performed to analyze the impact of removing each component on the model’s performance. The study included experiments conducted on both the two-DCS and four-DCS datasets involving the following scenarios:

1. Full working model with all components intact.
2. Full model without utilizing the spatial transformation network.
3. Model without the spatial transformation network and the inverse blur kernel estimation network, operating only the core pipeline using the ambiguous depth map as input.
4. Model with the spatial transformation network but without the inverse blur kernel estimation network, operating only the core pipeline using a grayscale image instead of the ambiguous depth map as input.
5. Model without the spatial transformation network and the inverse blur kernel estimation network, operating only the core pipeline using a grayscale image instead of the ambiguous depth map as input.

The experiment results summarized in Table 3 highlight the primary improvements in system performance. The most significant enhancement is observed when utilizing the ambiguous depth map as input, as employing grayscale led to notably higher RMSE and MAE values. Moreover, the model’s performance is substantially improved when the STN and ASIPM modules are used in conjunction. Removing these modules increased depth errors, particularly evident with the four-DCS datasets. Additionally, the model’s performance with two-DCS surpasses that with four-DCS, which was expected due to the lower blurriness noise in the input and fewer synchronization issues between the ambiguous depth map and grayscale. This enabled the STN to operate more effectively.

We carried out another activity to study the impact of continual learning. We compared the training results of the model using the static dataset followed by the dynamic dataset versus training the system using the dynamic dataset directly and without any static dataset. To perform this activity, we trained the system with different configurations:

1. For the normal case (i.e., the static dataset followed by the dynamic dataset), we used the default training configuration as described in Section 5.1.
2. For the dynamic dataset only, we used the training configurations as follows: using the Adam optimizer, the values of $\beta_{1}$ and $\beta_{2}$ were 0.9 and 0.999, respectively. The learning rate was scheduled to decay using the stepwise algorithm, where the starting rate was 0.0007 and the minimum rate was 0.0003. We performed 30 epochs but with a stepwise decay of 10 steps. We used dropout with a 25% rate and a batch size of 16.

**Figure 14:**
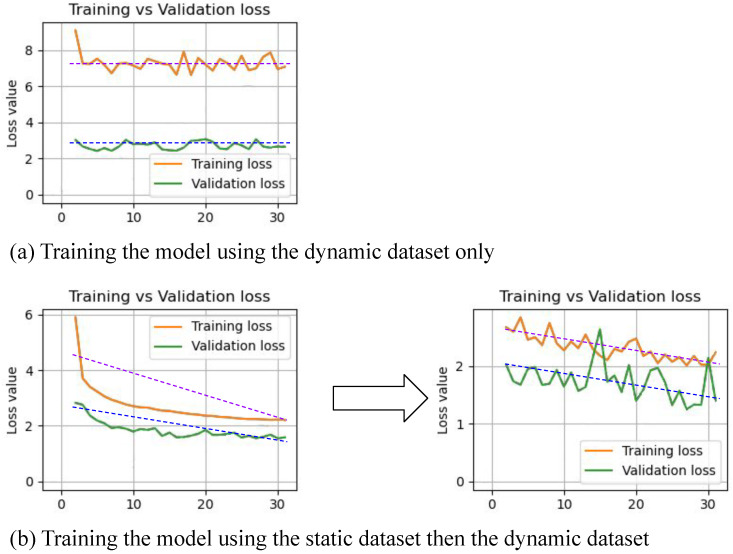
The diagram compares the training performance using (**a**) the dynamic dataset only, versus (**b**) the static dataset followed by the dynamic dataset. The diagram shows that the learning curve converges better when using the static dataset followed by the dynamic dataset, compared to using the dynamic dataset only.

In Figure 14, two cases are compared: (a) a learning process that remains stagnant without improvement, and (b) a successful training process using the static dataset followed by the dynamic dataset. Case (b) demonstrates that pretraining the model on static scenarios improves performance and learning curve progress in dynamic scenarios. This improvement supports the hypothesis that the model benefits from static scenario predictions relevant to certain dynamic situations. The proposed continual learning method contributes to improved performance in dynamic scenarios.

## 6. Discussion

This paper introduced a method to predict unwrap iTOF Lidar’s ambiguous depth maps in dynamic environments affected by motion blur and frame mis-synchronization. The method can predict unwrapped depth maps with reduced motion blur effects. The study investigated the hypothesis that training the model in static scenarios can improve its performance in dynamic scenarios by addressing motion blur. The proposed method incorporates the STN module for synchronization correction and the ASIPM module for predicting the inverse blur kernel tensor. The model is trained semi-supervised, reducing reliance on noise in the ground truth. Experimental results and ablation studies validate the hypothesis, with the model even outperforming the ground truth in some instances. These findings underscore the proposed model’s and training framework’s effectiveness in mitigating depth prediction issues inherent in the ground truth. In our future work, we plan to use a synchronized, better-performing sensor to generate the ground truth with accurate depth maps and no motion blur effect. We also plan to extend our dataset to include more postures of the sensor mounting on the ego vehicle. In addition, we plan to conduct sensor power measurements to study the impact of using only two-DCS frames on power reduction.

## Figures and Tables

**Table 1:** Comparative analysis between our method and closest state-of-the-art methods on the outdoor static dataset.

Method	Input	RMSE (m)	AbsRel	SqRel
**Modified Wild ToFu**	**iToF Wrapped Depth Map** **(4-DCS + GS)**	1.85	1.57	0.7
**Ours**	**iToF Wrapped Depth Map** **(4-DCS + GS)**	1.07	0.23	0.42
**Ours**	**iToF Wrapped Depth Map** **(2-DCS + GS)**	1.12	0.25	0.43

**Table 2:** Quantitative analysis for the results of the method using different datasets.

Dataset		Prediction Accuracy	Correction Accuracy	RMSE (m)	iRMSE (1/m)	SqRel	AbsRel	SiLog	MAE (m)	iMAE (1/m)
**Two-DCS**	**Static**	99%	99%	1.12	0.31	0.43	0.26	0.17	0.28	0.043
	**Dynamic**	89.69%	89.45%	3.89	0.29	0.021	0.012	0.43	2.31	0.0017
**Four-DCS**	**Static**	99%	99%	1.08	0.31	0.42	0.24	0.1	0.25	0.05
	**Dynamic**	89.74%	89.13%	4.02	0.3	2.33	1.32	0.46	2.42	0.0024

**Table 3:** Ablation study analysis between different implementations of our method.

		Prediction Frame		Correction Frame		RMSE (m)	iRMSE (1/m)	SqRel	AbsRel	SiLog
		Accuracy	Top 90% Precision	Accuracy	Top 90% Precision					
**Two-DCS**	**Full Mode**	89.69%	98.69%	89.45%	98.81%	3.88	2.92	0.021	0.012	0.42
	**W/o STN**	88.74%	98.71%	88.61%	98.88%	4.16	3.29	0.029	0.016	0.98
	**W/o Inverse** **Blur**	87.74%	98.63%	87.41%	98.72%	4.35	3.94	0.030	0.017	1.05
	**Using GS** **Only**	71.50%	99.17%	71.48%	99.17%	7.22	8.53	0.210	0.086	6.16
	**Using GS** **w/o STN**	71.36%	99.18%	71.33%	99.18%	7.2	8.53	0.220	0.087	6.17
**Four-DCS**	**Full Mode**	89.47%	98.43%	89.13%	98.81%	4.03	2.99	0.023	0.013	0.46
	**W/o STN**	84.70%	98.50%	85.18%	99.02%	4.76	4.14	0.043	0.022	1.77
	**W/o Inverse** **Blur**	86.23%	98.10%	87.05%	99.00%	4.38	3.45	0.028	0.016	0.70
	**Using GS** **Only**	71.36%	99.18%	71.35%	99.18%	7.20	8.53	0.220	0.086	6.17
	**Using GS** **w/o STN**	79.30%	97.88%	78.58%	98.22%	5.94	5.17	0.082	0.041	2.44

## Data Availability

Dataset is available upon request.

## Abbreviations

The following abbreviations are used in this manuscript:

MDPI	Multidisciplinary Digital Publishing Institute
iToF	Indirect Time-of-Flight
Lidar	Light Detection and Ranging
NFL	Near-Field Lidar
AMCW	Amplitude-Modulated Continuous Wave
CMOS	Complementary Metal-Oxide Semiconductor
ASIPM	Atrous Spatial Inception Pyramid Module
DCS	Differential Correlation Samples
SNR	Signal Noise Ratio
FPP	Fringe Projection Profilometry
STN	Spatial Transformer Network
GS	Grayscale
RMSE	Root Mean Square Error
MAE	Mean Absolute Error
SqRel	Squared Relative Difference
AbsRel	Absolute Relative Difference
SiLog	Scale-Invariant Logarithmic Error
W/o	Without
